# Assessment of 2′-Fucosyllactose and Lacto-N-Neotetraose Solution as an Irrigant in *E. faecalis*-Infected Root Canals: An In Vitro Study

**DOI:** 10.3390/clinpract14040108

**Published:** 2024-07-10

**Authors:** Francesco Puleio, Rosario Pirri, Vincenzo Tosco, Angelo Sergio Lizio, Paola Tripodi, Isabella La Spina, Vincenza La Fauci, Raffaele Squeri

**Affiliations:** 1 Department of Biomedical and Dental Sciences and Morphofunctional Imaging, Messina University, 98100 Messina, Italy; rosariopirri97@gmail.com (R.P.); angelolizio@libero.it (A.S.L.); paola.tripodi92@gmail.com (P.T.); isabella.laspina@gmail.com (I.L.S.); vlafauci@unime.it (V.L.F.); raffaele.squeri@unime.it (R.S.); 2 Department of Clinical Sciences and Stomatology (DISCO), Università Politecnica delle Marche, 60126 Ancona, Italy; v.tosco@pm.univpm.it

**Keywords:** endodontic irrigation, *Enterococcus faecalis*, human milk oligosaccharides, endodontic treatment

## Abstract

Background: Given the lack of an ideal endodontic irrigant on the market, this study evaluates the antimicrobial potential of a formulated solution of 2′-fucosyllactose and lacto-N-neotetraose against *E. faecalis* within infected root canals, and explores any associated impacts related to the duration of irrigation. Methods: 32 single-rooted teeth extracted for periodontal reasons were infected with *Enterococcus faecalis*, and subsequently subjected to endodontic treatment with two different irrigation systems: sodium hypochlorite or a solution of 2′-fucosyllactose and lacto-N-neotetraose. These samples were then incubated in sterile culture media at 37 °C to observe microbial activity through turbidity. The culture broth of each individual sample was assessed as positive or negative by observing the turbidity or lack of turbidity in the culture at the time of evaluation. Results: the analysis of the results obtained from the comparison of groups irrigated with sodium hypochlorite or a solution of 2′-fucosyllactose and lacto-N-neotetraose demonstrates that the case solution has no bactericidal effect against *E. faecalis* inoculated in the endodontic system. Conclusions: the HMOs used in this study do not have a bactericidal effect on *E. faecalis* inoculated in an endodontic system.

## 1. Introduction

The irrigation of the root canal system is crucial for achieving clinical success in endodontic therapy [1,2]. An ideal endodontic irrigant should possess characteristics such as high antimicrobial potency, the ability to denature and dissolve the smear layer, and high wettability to ensure sufficient contact with the canal walls [3]. Currently, there is no commercially available irrigant that encompasses all these features. Commonly used endodontic irrigants include sodium hypochlorite (NaClO), ethylenediaminetetraacetic acid (EDTA), chlorhexidine, and hydrogen peroxide [3,4,5,6].

NaClO exhibits potent antimicrobial activity by forming hypochlorous acid (HClO), which has a solvent effect on organic components [7]. EDTA, a calcium chelator, is used to denature and remove the smear layer rich in tissue residues and bacteria, acting specifically on canal hydroxyapatite without antimicrobial activity. It enhances the bactericidal effect of other irrigants and facilitates instrument movement during shaping phases [8]. Chlorhexidine is a broad-spectrum antimicrobial substance effective against Gram-positive, Gram-negative, and yeast [9]. Hydrogen peroxide releases hydroxyl ions (OH-) at low concentrations, resulting in a significant reduction in bacterial load [6]. However, the reported risk of oxygen gas release causing emphysema and the limited tissue lytic activity of chlorhexidine restrict their use in daily endodontic practice, making NaClO and EDTA the most commonly used irrigants [10,11,12]. Current irrigation protocols involve alternating NaClO (to attack organic residues and bacteria) and EDTA (to denature the mineral component and the smear layer produced by instrumentation), with intermediate irrigation phases using saline solution [8,13].

Considering endodontic treatment as an intervention aimed at addressing a microbiological issue, it is crucial to focus research on identifying more effective irrigants than those currently available. Bacterial persistence in the root canal post-treatment represents the primary cause of therapy failure [14,15,16,17]. Sodium hypochlorite, in particular, cannot eliminate all bacteria responsible for the infectious process [18]. Additionally, bacteria, aggregated in biofilm communities, exhibit interspecific interactions that increase resistance factors against antimicrobials, slowing down or inhibiting their action [19]. Endodontic therapy has a success rate of approximately 85–95% [20].

Researchers investigated the use of alternative irrigants to those currently used, such as the alcoholic extract of liquorice, Miswak, the essential oil of *L. sidoides*, the extracts of Neem, Tulsi, Bakul and Giloy, the Ayurvedic preparation known as ‘Triphala’, green tea polyphenols, propolis, and extracts of tropical fruits such as passion fruit [21].

Human milk has bioactive components that not only counteract bacterial proliferation but also promote the implantation of bacterial species producing infection resistance and immunomodulation factors [22].

Breast milk is a crucial nutrient for the development of the normal gut microbiota, protecting the newborn also from non-gut viruses such as the coronaviruses MERS-CoV and SARS-CoV-2 and the respiratory syncytial virus [23,24].

More than five thousand peptides synthesised from β-casein, immunoglobulin receptor and α-lactalbumin have been identified in breast milk, resulting from a pH-mediated selective proteolysis process [25,26]. They overlap in their aminoacid sequence with peptides that exert antimicrobial action [27].

The bioactive factors present within breast milk thus enable the formation of the infant’s first immune barrier in a healthy environment, given the vulnerability of the gastrointestinal system typical of this age [28].

Recent research has explored the antimicrobial potential of Human Milk Oligosaccharides (HMO) [29,30,31,32]. HMOs are molecules composed of five carbohydrate residues with a degree of polymerization ranging from 3 to 32. They can be classified into three broad groups, depending on the structure:

1. Fucosylates (35–50%);
2. Neutral non-fucosylates (42–55%);
3. Sialylates (12–14%) [33].

Found in breast milk, HMOs are ingested by the infant during breastfeeding and remain undigested in the infant’s intestine. The non-digestion of these oligosaccharides allows them to reach the developing intestinal bacterial flora, inhibiting the multiplication of some pathogenic species to promote the proliferation of beneficial bacteria for the child [20]. Moreover, their binding to pathogenic microorganisms prevents their adhesion to the intestinal epithelium [34].

Numerous studies have examined the antimicrobial effects of HMOs in vitro, demonstrating that HMOs at a concentration of 1–2 mg/L possess antimicrobial capabilities against group B streptococci [35]. Other studies have shown antimicrobial effects against *E. coli*, *C. jejuni*, *P. aeruginosa*, *S. enterica serovar fyris*, *S. dysenteriae*, *A. baumanii*, *H. pylori*, *V. cholerae*, *S. aureus*, and *E. faecalis* [36,37]. Furthermore, the contact with HMOs causes alterations in the cellular aggregation of group *B Streptococcus*, attributed to the saturation of carbohydrate transport systems, disrupting quorum sensing, and thus inhibiting biofilm formation [38]. Finally, a recent review demonstrated antiviral effects of HMOs due to complex HMO–viral protein interactions for viruses such as respiratory syncytial virus and human immunodeficiency virus (HIV) [39].

Therefore, due to the potential capabilities of HMO as a possible endodontic irrigant, the objective of this research is to verify the antimicrobial effectiveness of a titrated solution of 2′-fucosyllactose and lacto-N-neotetraose on *E. faecalis* inoculated in root canals and assess a possible correlation between the antimicrobial effect and irrigation time.

The null hypothesis is the lack of bactericidal effect of the case solution, with turbidity of all tested samples.

## 2. Materials and Methods

### 2.1. Study Design

This is a case–control study. Two analysis protocols were applied in this research. One protocol assessed the antimicrobial effectiveness of the case solution by comparing it with a control solution. The second protocol investigated the correlation between the antimicrobial effect of the case solution and the irrigation time of the samples, comparing two case groups with a control group.

### 2.2. Identification, Culturing, and Preparation of the Bacterium

The *Enterococcus faecalis* used in this study was identified through the SLIDE system (Liofilchem, Teramo, Italy) at the ‘G. Martino’ University Hospital in Messina. This system consists of a container containing a culture medium specific for Enterococci. In order to obtain a pure bacterial suspension without external contamination, a strain of *Enterococcus faecalis* was identified using Vitek2 compact 15 (BioMérieux Italia, Firenze, Italy).

Following identification, a quantitative and qualitative seeding was performed on a plate of Bile Esculin Azide Agar (BEA), a selective medium for enterococci. The isolated colonies were placed in a thioglycollate USP medium culture for 24 h at a temperature of 37 °C, resulting in a bacterial concentration of 3 McF. To obtain two suspensions at different concentrations, part of the 3 McF concentration suspension was diluted with additional sterile culture medium, resulting in a second suspension at a concentration of 1 McF. Thus, there are two bacterial suspensions at different concentrations: 1 and 3 McF.

### 2.3. Preparation of the HMO Solution

In this study, a solution containing HMO was utilized. The solution was obtained by diluting a commercially available dietary supplement (Nancare HMOs, Nestlé, Suzhou, China). According to the manufacturer’s instructions, 3 mL of the solution contains 500 mg of 2′-fucosyllactose (2′-FL) and 250 mg of Lacto-N-neotetraose (LnNT) dissolved in sterile physiological saline. The initial solution contained a total of 750 mg of HMOs. To achieve the target concentration of 20 mg/mL, dilution was necessary. The concentration calculation, defined as the ratio of the substance amount in milligrams to the solution volume in milliliters, indicated that dividing 750 mg by 20 mg/mL resulted in a final volume of 37.5 mL. After subtracting 3 mL to account for the initial HMO volume, we added 34.5 mL of sterile saline. Thus, the final solution comprised 20 mg/mL of HMOs dissolved in 37.5 mL of sterile saline.

### 2.4. Sample Preparation

In this study, 32 single-rooted teeth extracted for periodontal reasons were selected. The sample size was determined by considering two study groups receiving different treatments. We assumed a success probability of 95% in the control group. For the treated group, the desired success rate was set at 20. The analysis aimed to detect this substantial difference with a power of 0.85 and a significance level set at 0.05, which are standard parameters for ensuring robust study outcomes. This setup required a sample size of approximately 6 subjects per group to achieve the desired statistical power.

The selected teeth were decrowned at 15 mm from the anatomical apex using a diamond bur (6837 KR Komet). The samples were sterilized in an autoclave (Domina Plus B Premium, Dental X, Vicenza, Italy) using a steam sterilization protocol (25 min × 132–134 °C). Canal patency was confirmed with a size 10 K-file. The glide path was created using a ProGlider instrument (Dentsply Maillefer, Ballaigues, Switzerland) at 300 RPM and 2 Ncm. The root canals were then shaped with ProTaper Next^®^ X1, X2, and X3 (Dentsply Maillefer, Ballaigues, Switzerland) at 300 RPM and 4 Ncm. During the shaping phase, the instruments were taken 1 mm beyond the apex. After using each endodontic instrument, the canals were irrigated with 1 mL of 5% NaOCl using a 30 G needle positioned at the apical foramen, and the irrigant was activated with sonic activation (Endoactivator^®^, Dentsply Maillefer, Ballaigues, Switzerland). Following the shaping phase, irrigation with 5 mL of 17% EDTA was performed using a 30 G needle positioned at the apical foramen, delivered at a rate of 5 mL/min. Each canal was subsequently irrigated with 5 mL of deionized water and dried with paper points.

Each sample was randomly divided into 5 groups.

Group A (Case Group) *n* = 7: **
  - *E. faecalis* was inoculated into the canal at a concentration of 3 McF.
  - The canal of each sample was irrigated with 5 mL of the case solution containing HMO using a 30 G needle positioned at the anatomical apex. The HMO solution was delivered at the rate of 1 mL/30″. An extracanal lavage was performed with the same syringe, followed by canal irrigation with physiological saline. The canals were dried with paper points.
Group B (Control Group) *n* = 7: **
  - *E. faecalis* was inoculated into the canal at a concentration of 3 McF.
  - The canals of the samples were irrigated with 5 mL of 5% NaOCl using a 30 G needle positioned at the apical foramen. The irrigant was activated with sonic activation (Endoactivator^®^, Dentsply Sirona, Ballaigues, Switzerland). An extracanal lavage was performed with the same syringe, followed by canal irrigation with physiological saline. The canals were dried with paper points.
Group C (Case Group) *n* = 6: **
  - *E. faecalis* was inoculated into the canal at a concentration of 1 McF.
  - The canal of each sample was irrigated with 3 mL of the case solution containing HMO using a 30 G needle positioned at the anatomical apex. The HMO solution was delivered at the rate of 3 mL/1″. An extracanal lavage was performed with the same syringe, followed by canal irrigation with physiological saline. The canals were dried with paper points.
Group D (Case Group) *n* = 6: **
  - *E. faecalis* was inoculated into the canal at a concentration of 1 McF.
  - The canal of each sample was irrigated with the case solution using a 30 G needle positioned at the anatomical apex. The HMO solution was delivered at the rate of 3 mL/2″. An extracanal lavage was performed with the same syringe, followed by canal irrigation with physiological saline. The canals were dried with paper points.
Group E (Control Group) *n* = 6: **
  - *E. faecalis* was inoculated into the canal at a concentration of 1 McF.
  - The canals of the samples were irrigated with 1 mL of 5% NaOCl using a 30 G needle positioned at the apical foramen, and the irrigant was activated with sonic activation (Endoactivator^®^, Dentsply Sirona, Ballaigues, Switzerland). An extracanal lavage was performed with the same syringe, followed by canal irrigation with physiological saline. The canals were dried with paper points.

The samples from each group were placed in individual sterile test tubes containing liquid culture medium (thioglycollate usp medium) and incubated at 37 °C. The culture broth of Group A (Case Group) and Group B (Control Group) samples was observed after 24 h of incubation at 37 °C. The culture broth of Group C (Case Group), Group D (Case Group), and Group E (Control Group) samples was observed after 24 and 36 h of incubation at 37 °C.

### 2.5. Statistical Analysis

The culture broth of each individual sample was assessed as positive or negative by observing the turbidity or lack of turbidity in the culture at the time of evaluation. The culture medium was observed by two different operators, and in the case of result discrepancies, a third expert operator’s evaluation was sought.

To assess the significance of differences between groups A and B, the Fisher’s exact test was applied. This statistical test is commonly used for analyzing categorical data and is appropriate for assessing the significance of associations between two categorical variables, such as the presence or absence of bacterial growth in this study. The Fisher’s exact test helps determine if there is a significant difference in the proportions of positive and negative cultures between the two groups. In addition to the Fisher’s exact test, the chi-squared test was also applied to further evaluate the associations between the categorical variables in our study. This statistical test is typically used to determine whether there is a significant difference between the expected frequencies and the observed frequencies in one or more categories of a contingency table. The results were interpreted at a 95% confidence level to determine if any observed differences were statistically significant.

## 3. Results

The culture turbidity results are grouped in Table 1, Table 2 and Table 3.

A total of 85.71% of the samples in group A were found to be turbid after 24 h of observation.

A total of 14.29% of the samples in group B were found to be turbid after 24 h of observation.

A total of 100% of the samples in group C were turbid after 24 and 36 h of observation.

A total of 100% of the samples in group D were not turbid after 24 h of observation but became turbid at the 36 h check.

A total of 100% of the samples in group E were not turbid at 24 and 36 h.

The difference between groups A and B was statistically significant (*p* < 0.05). The chi-square value is 4.57.

## 4. Discussion

Human milk oligosaccharides (HMOs) potentially have antimicrobial effects because they resemble the surface receptors of epithelial cells, acting as decoy receptors for certain bacterial species that adhere to the epithelium for colonization. This prevents bacterial biofilm formation by inhibiting adhesion. Additionally, HMOs may exert bactericidal effects by impairing bacterial metabolism. However, it is important to note that these effects are species-specific.

Despite the high success rate of current root canal irrigation protocols, irrigants may not completely eradicate microbial components from the canal system, especially in the presence of periapical lesions [40]. The identification of new irrigants could assist clinicians in improving canal disinfection.

This study represents the first evaluation of using Human Milk Oligosaccharides (HMO) as endodontic root canal irrigants. In a previous study, a bactericidal effect was achieved with a pool of HMO, including LnNT, at concentrations between 20 and 50 mg/mL [37]. Therefore, in this study, the HMO solution was prepared at the same concentration considered effective in the aforementioned study. 2′-FL lacks bactericidal effects [37].

The shaping of the canals was carried out for two reasons: the first, to allow better penetration of the solution containing the bacterium; the second concerns the possibility of simulating a reality as close as possible to that of the clinic, with *E. faecalis* reinfecting the shaped and cleaned endodontic system [41].

The microbiological examination in our study is qualitative and macroscopic. After applying the case or control irrigation protocols to the samples infected with *E. faecalis*, the samples were immersed in a culture broth; the proliferation of any remaining bacteria inside the canal system makes the culture broth turbid. Turbidity of the culture broth was used as a positive/negative indicator of the effectiveness of the irrigation protocols [42]. In this research, the potential bactericidal effect of the tested solution and the possible time-related effect on bacterial replication after being treated with HMOs were evaluated. The efficacy of the case solution in eliminating *E. faecalis* from root canals was analyzed by evaluating the turbidity of the culture medium in which the samples from group A were immersed and comparing it with the turbidity of the culture medium from group B, whose samples were irrigated with the control solution. Although *E. faecalis* has a doubling time of 22 ± 2 min, the samples were observed after 24 and 36 h of incubation to obtain an objective and macroscopically visible result. Therefore, after 22 min, the presence of the bacterium, if it were in low quantities, would not be appreciable macroscopically. Sodium hypochlorite was chosen as the irrigant for group B (control group); it is considered the most widely used root canal irrigant with well-documented efficacy in the literature [3,7,43,44]. The evaluation of the influence of irrigation time was performed by comparing the turbidity of the culture medium of groups C, D, and E at 24 and 36 h post-irrigation. Identification by culture selection and Vitek2 (automated) ensured the purity of the bacterial suspension, which was thus free of the presence of other bacterial species [45]. In this research, various bacterial concentrations were tested to simulate different severities of infection. The analysis of the results obtained from the comparison of groups A and B demonstrates that the case solution has no bactericidal effect against *E. faecalis* inoculated into the endodontic system. A total of 83.34% of the samples in group A tested positive at the 24 h check, indicating that the inoculated bacteria inside the samples were not eliminated and subsequently multiplied in the culture medium. Sodium hypochlorite used as the control solution in group B was effective in 83.34% of the samples. The Fisher’s exact test demonstrated a statistically significant difference between the results of group A and group B.

The analysis of turbidity in groups C, D, and E at 24 and 36 h showed no correlation between the antimicrobial effect of the case solution and the irrigation time of the samples. A total of 100% of the samples in group C, irrigated with HMO solution for 1 min, tested positive at both the 24 and 36 h checks; the samples in group D, irrigated with HMO solution for 2 min, tested negative at the 24 h check but subsequently tested positive at 36 h. The variation in turbidity of the culture medium in group D is attributable to a probable bacteriostatic effect of the HMO-containing solution, which blocked the proliferation of bacteria in the first 24 h. The samples in group E, irrigated with sodium hypochlorite used as the control group, tested negative at both 24 and 36 h.

Further research is needed into the bacteriostatic rather than bactericidal effect of root canal irrigants in order to establish the degree of intracanal bacterial survival. Further research should also evaluate the use of other HMO pools more similar to the composition of human milk; it would therefore be possible to re-test with different mixes, at different concentrations and contact times with the bacterium, both in planktonic and biofilm form.

No clinical indications can therefore be drawn from this research: the irrigant we tested should not be used as a root canal irrigant during endodontic therapy.

### Limitation

The first limitation of this study is related to the use of the McFarland Standard, which, by evaluating the turbidity of a culture broth, allows for an assumption about the presence and concentration of bacteria within the culture broth itself. However, it is not an exact indicator of the quantity of bacteria present in the culture. The result was thus evaluated using a dichotomous variable: turbid = positive, non-turbid = negative. Further studies should analyze the extent of bacterial inhibition by assessing the efficacy of the solution at different concentrations, along with various suspensions of bacterial concentrations.

The second limitation is that authors who have analyzed the antimicrobial effects of HMOs use a pool containing various HMOs because the antimicrobial effects of these substances are species-specific and challenging to attribute to a single HMO [35]. In this study, only one of the HMOs (lacto-N-neotetraose) was analyzed, certainly not representative of the chemical diversity of these compounds.

A final limitation of this research is that the HMO solution was tested on only one bacterial species (*E. faecalis*). To provide guidance on its potential use as an endodontic irrigant, it is necessary to test the molecule on mature biofilms of various species infecting the endodontium.

## 5. Conclusions

Within the limitation of this research, the analysis of the results obtained indicates that the HMOs used in this study do not have a bactericidal effect on *E. faecalis* inoculated in an endodontic system, verifying the null hypothesis. It is not clear from the results of this study whether the HMOs used may have bacteriostatic effects. Further studies are needed to assess the effectiveness of different types of HMOs.

Further tests should be conducted to verify the effect of HMOs on bacterial proliferation or eradication, with quantitative rather than qualitative assessments. Finally, the solution to be tested should contain a broader and more varied pool of HMOs to evaluate not only different molecules but also potential synergistic interactions.

## Figures and Tables

**Table 1:** Results of turbidity after 24 h of Groups A, B.

Group A	Group B
Positive	Negative
Positive	Negative
Negative	Negative
Positive	Negative
Positive	Positive
Positive	Negative
Positive	Negative

**Table 2:** Results of turbidity after 24 h of Groups C, D, E.

Group C	Group D	Group E
Positive	Negative	Negative
Positive	Negative	Negative
Positive	Negative	Negative
Positive	Negative	Negative
Positive	Negative	Negative
Positive	Negative	Negative

**Table 3:** Results of turbidity after 36 h of Groups C, D, E.

Group C	Group D	Group E
Positive	Positive	Negative
Positive	Positive	Negative
Positive	Positive	Negative
Positive	Positive	Negative
Positive	Positive	Negative
Positive	Positive	Negative

## Data Availability

The original contributions presented in the study are included in the article, further inquiries can be directed to the corresponding author.
